# Sex Differences in the Associations Among Early Life Adversity, Inflammation, and Cognition

**DOI:** 10.3390/biom15020161

**Published:** 2025-01-22

**Authors:** Erin Logue, Charles B. Nemeroff

**Affiliations:** 1 Department of Psychiatry and Behavioral Sciences, Mulva Clinic for Neurosciences, The University of Texas at Austin Dell Medical School, Austin, TX 78712, USA; 2 Department of Neurology, Mulva Clinic for Neurosciences, The University of Texas at Austin Dell Medical School, Austin, TX 78712, USA; 3 Institute for Early Life Adversity, The University of Texas at Austin Dell Medical School, Austin, TX 78712, USA

**Keywords:** early life adversity, cognitive decline, sex differences, adverse childhood experiences

## Abstract

Early life adversity (ELA) has long been recognized to negatively impact a variety of health outcomes, with increasingly recognized long-term implications for neurocognitive function. ELA may affect the brain through multiple mechanisms, including chronic inflammation. One potential moderator of the pathway from ELA to neuroinflammation to cognitive dysfunction is sex. ELA may leave females potentially even more vulnerable to cognitive impairment in later life. This review discusses the influence of ELA on cognitive function across much of the lifespan, how inflammation is implicated in this process, and the current state of knowledge regarding sex differences in these relationships. We conclude with a discussion of unanswered questions and suggestions for future research, including the incorporation of genetic data.

## 1. Introduction

Early life adversity (ELA) encompasses not only trauma and other adverse childhood experiences (ACEs) but also additional significant stressors such as financial hardship, neighborhood deprivation, and racism [1,2,3]. Data from the Behavioral Risk Factor Surveillance System indicates that between 2001 and 2020, greater than 63% of adults were exposed to at least one adverse experience in childhood, and 17.3% were exposed to four or more [4]. Recent data indicate that before age four, approximately one in five individuals have experienced adversity, with one in eight having at least two adverse experiences. These numbers increase when additional factors encompassing ELA are considered.

ELA has long been recognized as a risk factor for poor physical and mental health outcomes [5]. A meta-analysis conducted on adults with at least four ACEs revealed stronger associations with mental health outcomes (e.g., mental illness, drug and alcohol abuse, and suicide) than physical health outcomes (e.g., heart disease, cancer, and diabetes) [6]. Multiple mechanisms to account for these associations have been proposed, including the role of inflammation, and extensively discussed in previous reviews [2,7]. Increasingly, long-term negative impacts on cognition are also being recognized in a growing body of evidence, with most studies finding some relationship between ELA and cognition. However, sex is often not modeled in human studies on this topic. This review will therefore discuss the influence of ELA on cognitive function across the lifespan, how inflammation is implicated in this process, and the current state of knowledge regarding sex differences in these relationships.

## 2. Early Life Adversity’s Impact on Cognition

### 2.1. In Childhood and Adolescence

In children and adolescents, some of the most consistent and strongest findings regarding the impact of ELA on cognition are related to the effects of childhood maltreatment and other interpersonal traumas on increased risk for cognitive delay as well as low intelligence, attention, and executive function when compared to healthy controls [8,9,10,11,12,13]. Impacts on processing speed have also been found [11]. Findings from studies of memory in youth with trauma histories are inconsistent but generally show that trauma-exposed youth perform worse on measures of learning and memory than healthy controls [8,12,14]. These cognitive effects vary with the severity and number of posttraumatic stress symptoms and the duration of maltreatment [8,13]. Deprivation, including through institutionalization for extended periods, also confers risk for deficits on tests of intelligence and academic abilities as well as neuropsychological tests of attention, working memory, language, memory, inhibitory control, and other executive functions [15,16,17,18,19,20].

However, the connection between ELA and cognition is far from straightforward. The degree of these cognitive deficits may also relate to developmental timing or class of adversity (i.e., deprivation versus threat) [12,18,21]. Yet, findings regarding the onset of trauma related to cognitive outcomes in youth are variable [12,13]. Furthermore, some studies have found that cognitive ability prior to ELA largely accounts for the relationship between ELA and cognitive function [22], and recent evidence from a large longitudinal cohort suggests that mental health may mediate the association between early adversity and certain cognitive functions in later childhood [23]. Findings regarding cognitive outcomes in adults with ELA are similar and described below.

### 2.2. In Adulthood

Most studies conducted on this topic using data from longitudinal cohorts find a negative relationship between ELA and cognition in adulthood. Such studies have typically found that individuals with higher numbers of ACEs are more likely to experience cognitive dysfunction in both early adulthood and later in life [24,25,26,27,28,29]. Similarly, in a longitudinal cohort of females, more severe and more frequent sexual abuse was more strongly associated with lower scores on measures of cognition in mid-life [30]. The most consistent findings from these studies relate to memory and executive function, though psychomotor speed and attention are also implicated. Few studies report findings of better cognition related to ELA [31].

Cross-sectional studies indicate similar findings in terms of the domains impacted in adults. Examples include college students exposed to sexual abuse in childhood (poorer memory and inhibitory control as compared to health controls [32]), early and middle adults exposed to multiple ACEs (worse executive function [33]), and adults exposed to early food insecurity (poorer cognition [34]). However, cross-sectional studies have not always found a relationship between the number of ACEs and cognitive function [35]. Others present mixed findings, with some ACEs (e.g., physical abuse and sexual abuse) negatively impacting cognition and others exhibiting the opposite effect [36,37,38].

Other studies have found the effect of ELA on cognition mediated and moderated by additional factors. For example, education has regularly been cited as a mediating factor in the link between ACEs, early deprivation, socioeconomic disadvantage in early life, and cognitive outcomes in later life [25,39,40,41]. Other investigations have not found that demographic factors account for such findings [29]. Depression and frequent distress have been found to mediate the effect of ELA on cognition in adulthood, while familial risk for mental illness has been found to moderate it [42,43,44,45]. Another identified moderator is social isolation [46].

Studies using a life course stressor approach generally find evidence to support both stressors in childhood and adulthood (recent and remote) being associated with lower baseline levels of concentration, memory, processing speed, and/or executive function but have not always found evidence for a faster rate of decline [24,40,47]. In one longitudinal cohort study, a faster decline in cognition in older adulthood over a ten-year period was only observed for individuals with ACEs and depression, as opposed to ACEs and no depression [48]. Others have found steeper declines in only certain cognitive abilities related to early life adversity [28,49] or increasing numbers of deprivation-related ACEs but not threat-related ACEs [46]. One study found less decline in executive function in individuals with higher numbers of ACEs and no significant association between the number of ACEs and baseline cognitive function [50].

Finally, there are trends related to certain ELAs across studies. Maltreatment and abuse, poverty and socioeconomic disadvantage, food insecurity, and parental death most consistently show relationships or show the strongest relationships with cognitive outcomes [27,28,32,39,41].

### 2.3. Summary

Across the youth and adult literature, there are clear trends. One is a dose–response relationship, with increased severity, frequency, duration, and numbers of ACEs generally negatively related to cognitive outcomes. Other similarities include the notion that baseline cognitive function may play a mediating role in mental health factors. Few published studies have reported no relationship or a positive relationship between ELA and cognition in youth and adults. Differences in the child and adult literature include memory shown to be more consistently impacted in adult versus child samples.

## 3. Inflammation as a Potential Mediator

Questions remain as to the underlying biological processes that trigger brain changes leading to cognitive dysfunction in the ELA-exposed population. In fact, ELA may affect the brain through multiple mechanisms. This paper focuses on the mechanism of chronic inflammation. Although this topic has been extensively reviewed in this special issue as well as elsewhere [51], it deserves mention here as a segue into sex differences.

### 3.1. The Immune Response, Inflammation, and Hypothalamic–Pituitary–Adrenal (HPA) Axis Function

The immune response involves the binding of pathogen recognition receptors to not only pathogen-associated molecular patterns, such as those from viruses and bacteria, but also to endogenous danger/damage-associated molecular patterns (DAMPs) [52,53]. Thus, when stressors such as ELA produce excessive levels of stress hormones, this initiates a cascade of events that, as with pathogens, leads to the recruitment and activation of various immune cells [53,54]. This process is mediated by inflammatory cytokines and chemokines [55].

The HPA axis plays a crucial role in modulating inflammatory responses through its primary effector, cortisol. Upon activation by various stressors, including inflammatory stimuli, the hypothalamus releases corticotropin-releasing hormone (CRH), which stimulates the anterior pituitary to secrete adrenocorticotropic hormone (ACTH). ACTH then triggers the adrenal cortex to produce glucocorticoids, primarily cortisol [56]. Cortisol exerts potent anti-inflammatory effects by suppressing proinflammatory cytokine production, inhibiting leukocyte trafficking, and promoting the resolution of inflammation [57]. This feedback mechanism serves to prevent excessive or prolonged inflammatory responses that could lead to tissue damage or chronic inflammatory conditions. However, prolonged or repeated activation of the HPA axis, as seen in a chronic stress situation such as ELA, can lead to glucocorticoid resistance and dysregulation of the inflammatory response, potentially contributing to the development of various inflammatory disorders [58].

Conversely, inflammatory processes can significantly influence the HPA axis function, creating a bidirectional relationship between these two systems. Proinflammatory cytokines, such as interleukin-1β (IL 1-β), tumor necrosis factor-α, and interleukin-6 (IL-6), can activate the HPA axis at multiple levels, including direct stimulation of CRH and ACTH release [59]. As indicated above, although this is an important adaptive response to acute inflammation, in conditions of chronic inflammation, persistent cytokine elevation can lead to HPA axis dysregulation, characterized by blunted cortisol responses to stress and aberrant reactivity [60,61]. This dysregulation can further exacerbate inflammatory processes, creating a potential feedback loop that may contribute to the pathogenesis of various stress-related and inflammatory disorders [62].

### 3.2. Evidence for ELA’s Relationship to Inflammation

Multiple early life stressors have been shown to lead to inflammation through both indirect and direct means. This includes childhood trauma. In one study, ACEs at age 7 years were positively associated with IL-6 at age 10, and ACEs at age 8 were positively associated with CRP at age 15 after adjusting for CRP at age 10 [63]. However, a meta-analysis on the relationship between ELA and inflammation in children and adolescents found small effect sizes for CRP and IL-6 [64]. The authors cautioned that the small number of studies included for analysis were highly heterogeneous.

Childhood trauma has been variously linked to inflammation in adulthood. A meta-analysis of 16,870 participants found that compared to adults without a history of childhood trauma, those with such a history had significantly higher levels of C-reactive protein (CRP), TNF-alpha, and IL-6 [65]. An updated meta-analysis did not find an association between childhood trauma and TNF-alpha and found differential links between certain childhood traumas and CRP and IL-6 [66]. The authors noted the potential for other variables at play, with some populations at higher risk for chronic inflammation, and suggested sex as a potential mediator worthy of future investigation. The strength of these associations also appears to increase over time across various early life adversities [65].

Specific forms of childhood adversity have also been tied to elevated inflammatory markers. Norton found a significant association between childhood experiences of family death and elevated levels of high-sensitivity CRP in later life [67]. Additionally, adolescents exposed to greater levels of peer victimization demonstrated increased levels of IL-6 and IL-1β [68]. Other studies have suggested that victims of different types of abuse show varied inflammatory responses: physical abuse was associated with IL-2R, sexual abuse with IL-1β, and emotional abuse with IL-6 [66,69,70,71]. Adolescents exposed to community violence, observed in neighborhoods with high crime rates and poverty, evidenced higher levels of the proinflammatory markers CD14/CD16 in comparison to peers living in more stable environments [72]. Similar to peer victimization findings, the accumulation of social adversities, such as financial hardship, correlated with increased levels of CRP and other inflammatory markers [73]. Collectively, these findings highlight the links between ELA and inflammation and suggest that the biological embedding of stress can have lasting consequences on health through mechanisms involving inflammatory processes.

### 3.3. Impact on Cognition

Damaging states caused by prolonged stress include neuroinflammation and dysregulated brain network integrity [65,74,75,76]. Dysregulation of the HPA axis resulting from maltreatment impacts brain development, including in the prefrontal cortex and hippocampus, and neuroinflammation may be linked with cognitive impairment in later life, partly through altered adult hippocampal neurogenesis [51,77,78]. Indeed, inflammation has been shown to play a mediating role in the relationship between ELA and cognitive function in later life, especially those involving prefrontal and hippocampal processes, in cross-sectional investigations [79]. For example, Davis et al. found that IL-6 mediated the negative relationship between childhood abuse and global cognitive performance in later life [80]. Allostatic load, of which inflammatory cytokines are components, has also been shown to mediate the relationship between ELA and executive function in females in later life [81]; however, a prospective cohort study did not find this mediation effect [82].

### 3.4. Summary

Findings suggest that although the inflammatory response initiated by early adversities may disrupt neurodevelopmental processes crucial for cognitive functioning, additional variables are likely at play. One potential moderator is sex.

## 4. Sex Differences

### 4.1. In Cognitive Outcomes Following ELA

Many studies on the impact of ELA on cognition in adulthood do not model sex differences. Nevertheless, some evidence suggests that the impact of ELA on cognitive outcomes may differ between adult males and females. Studies that explicitly model sex differences have found that females who experience ELA, including abuse, demonstrate worse processing speed and delayed recall scores than males [27,41,45]. A longitudinal cohort study found that females raised in households with socioeconomic disadvantage exhibited more accelerated decline in delayed recall than males [41]. Another study found that ELA worsened performances on measures of learning for females but improved performance for males [83]. Wang et al. found that ACEs were more detrimental to females than males in that they increased depression symptoms and decreased cognitive function for each additional ACE experienced [45]. Some studies have modeled sex but not the interaction between sex and ELA; therefore, although such studies may report on whether there is a main effect of sex on cognition, how this interacts with trauma remains unknown. One exception was a longitudinal study that found no interaction between ACEs and sex in predicting memory performance or decline over time [47].

Although the above findings, taken together, suggest that adult females may be more vulnerable to the cognitive consequences of ELA, particularly in the learning and memory domain, research in youth indicates either no difference between male and female children [84] or that young males may be more susceptible to certain cognitive impairments following ELA. In a study examining the effect of institutionalization on cognition in childhood, sex did not modify the main effect of group on cognition [18]. In a meta-analysis on the effect of trauma exposure on executive function in youth, sex was not a moderator. Neither did sex interact with exposure to intimate partner violence between parents to predict memory performance in children [85]. The finding of lower IQ and visual memory among youth exposed to abuse and neglect versus those not exposed did not differ by sex in another study [86]. Yet, a longitudinal study found that boys from low-income backgrounds showed greater declines in working memory capacity during childhood compared to girls from similar socioeconomic circumstances [87]. Similarly, a meta-analysis found that there was a smaller effect of threat ACEs on working memory in children and adolescents in studies with a greater proportion of females [21].

These findings highlight the complexity of sex differences in the relationship between ELA and cognitive outcomes, suggesting that vulnerability may depend on the specific type of adversity, the cognitive domain being assessed, and the developmental period of those being assessed.

### 4.2. In Immune and Inflammatory Response

Largely due to the influence of sex hormones and evolutionary adaptations related to reproduction, the female immune system exhibits several distinct characteristics compared to that of males. The X chromosome also plays a crucial role in shaping the female immune system. Many genes involved in immune function are located on the X chromosome, and the presence of two X chromosomes in females can lead to increased expression of these immune-related genes. Furthermore, the process of X-chromosome inactivation, where one X chromosome is randomly silenced in each cell, can create a mosaic effect in females, potentially providing a broader range of immune responses. The generally higher immune reactivity in females is also attributable to the effects of estrogen, which stimulates the production of antibodies and promotes the activity of various immune cells [88,89]. As a result, females typically mount stronger and faster immune responses to pathogens and vaccines.

Another difference lies in the cyclical nature of the female immune system, which fluctuates throughout the menstrual cycle and with pregnancy and menopause. For example, during the follicular phase of the menstrual cycle, when estrogen levels are high, the immune system is more active and responsive. Estrogen’s effect on the immune system must also be considered within the greater hormonal environment and state of the body. The female immune system undergoes significant changes during pregnancy, mediated by increases in estrogen and progesterone (a natural immune suppressor), to prevent rejection of the fetus while maintaining protection against pathogens. This state is associated with a decrease in proinflammatory cytokines. Contrary to this, the decline in estrogen during menopause can lead to weakened immunity and increased inflammatory markers [90,91].

Heightened immunity, genetic differences, and genetic diversity offer females greater protection against and an evolutionary advantage in fighting off a wider variety of pathogens but also increase their susceptibility to inflammatory illnesses [92]. Furthermore, the cyclic variation in immune function can affect a woman’s susceptibility to infections and immune-related illnesses at different points in her menstrual cycle and her life.

### 4.3. In Relationships Among ELA, Inflammation, and Cognition

Although the Baumeister et al. meta-analysis did not find a moderating effect of sex on the relationship between ELA and inflammatory markers, when sex differences are found in the relationships among these factors, they are found for females [65]. Two studies found a longitudinal association between ELA and CRP for females versus men [92,93]. Furthermore, in the D’Amico et al. study, the mediating role of allostatic load in the relationship between ELA and executive function was found only for females [81].

Even though inflammatory markers are only one component of allostatic load, mechanisms underlying any sex differences in ELA-related cognitive dysfunction may involve complex interactions between sex hormones, stress hormones, the inflammatory response, and neurodevelopmental processes [81,94]. Doom and Gunnar suggested that sex differences in stress reactivity and regulation during different points in development might contribute to differential vulnerability to ELA-induced cognitive dysfunction, noting how the stress response between the sexes becomes more pronounced at puberty [95]. Furthermore, while estrogen generally enhances immune function, leading to increased reactivity and production of inflammatory cytokines, estrogen also has neuroprotective effects [96]. The decline of estrogen during menopause may increase the risk of cognitive decline, a process potentially compounded by a brain already vulnerable due to the cognitive effects of early life adversity [97]. Thus, ELA may leave females potentially even more vulnerable to cognitive impairment and developing neurocognitive disorders in later life.

## 5. Conclusions and Future Directions

The picture that seems to come together based on all of the above-reviewed literature is that individuals assigned male at birth may be more vulnerable to the cognitive effects of ELA in childhood, whereas the effects for females become more pronounced with time. Females appear to be more vulnerable in later life, especially when considering effects on memory. This may be due to differential immune and inflammatory systems of females and associated with a proposed incubation effect, in which there is a prolonged glucocorticoid response to stressors experienced during adolescence that is not fully manifested until adulthood [98].

Indeed, chronic inflammation has been tied to ELA. Neuroinflammation, which can be caused by chronic inflammation, has been associated with cognitive decline and neurodegenerative diseases such as Alzheimer disease (AD), which is more common in females [99]. Although this is likely for a variety of factors, declines in estrogen during menopause and heightened immune reactivity in females could potentially contribute to increased neuroinflammation under certain conditions, including ELA. However, unanswered questions remain.

For example, the literature reviewed suggests that different types of ELA (e.g., abuse, neglect, and socioeconomic adversity) might have distinct cognitive and inflammatory outcomes. Further research is required to further delineate these differences and investigate them by sex, potentially leading to more targeted and effective interventions. More longitudinal studies are needed to track relationships between ELA, inflammation, and cognitive dysfunction over time to examine how they evolve based on age and sex. This requires not only systematically capturing different dimensions of ELA but also jointly and consistently assessing multiple domains of cognition. For example, if studies confirm that males are more vulnerable to cognitive effects of ELA in childhood and these are found to be most pronounced with deprivation, whereas females show more pronounced effects later in life, particularly on memory and when they have a history of abuse as children, this could inform the timing and focus of interventions. Clinicians and policy makers might prioritize early cognitive support and resilience-building programs for boys who have experienced ELA while directing long-term monitoring and memory-focused interventions to the most at-risk females with a history of ELA.

Understanding how genetic vulnerability to cognitive decline or epigenetic changes might influence the ELA–cognition relationship, particularly in how they interact with sex and inflammation, could also provide new avenues for personalized medicine. For example, worse attention and working memory have been found in females with childhood trauma and the *APOE* ɛ*4* allele versus those with the *APOE* ɛ*4* allele or a history of childhood trauma alone [100]. *APOE* ɛ*4*, codes for a protein associated with higher risk for developing AD (particularly in females), causes an increased proinflammatory response through heightened glial activity [99,101]. A question remains as to whether ELA contributes to this inflammatory cascade synergistically. The *FKBP5* gene is also proinflammatory, and its expression increases with age. This gene regulates glucocorticoid receptor function and encodes FKBP51, the latter of which regulates tau (another protein implicated in AD) [102]. At least in mice, *FKBP5* expression is also increased with early life adversity and may have a sex-dimorphic influence [102,103]. This gene has also been shown to interact with early life adversity to increase a host of psychiatric symptoms in humans [104,105]. If studies confirm a synergistic effect between ELA and genetic factors like *APOE* ɛ*4* or *FKBP5* on inflammation and cognitive decline, it could lead to targeted screening programs for individuals with both ELA history and genetic risk factors. This could inform early intervention strategies, such as cognitive training or medicines, tailored to an individual’s specific risk profile in which sex may be a factor.

Finally, although this paper focused on sex differences, it is important to draw attention to the fact that research aimed at including gender-diverse individuals (i.e., people who identify with a gender other than that the sex assigned at birth) at the intersection of ELA, inflammation, and cognition is also lacking. This is despite emerging findings indicating that gender-diverse individuals experience increased ACEs [106,107]. It is therefore recommended that future research incorporate gender identity in addition to sex assigned at birth as a variable of interest. This could help provide for more inclusive and comprehensive interventions, prompting the development of specialized support services and policy changes to address the unique needs and vulnerabilities of this population.

Ultimately, further understanding of sex- and gender-specific differences in the link between early life adversity, inflammation, and cognition is crucial for developing targeted interventions and support strategies for all individuals who have experienced early life adversity.

## Data Availability

Not applicable.
